# Supplementary material for: Screening of Metabolism-Disrupting Chemicals on Pancreatic α-Cells Using In Vitro Methods
Source: Int J Mol Sci. 2022 Dec 23;24(1):231. doi: 10.3390/ijms24010231 (PMC9820113; doi:10.3390/ijms24010231)
Supplement: Supplementary file 1 [file ijms-24-00231-s001.zip › ijms-2066646-supplementary.pdf]

Supplementary Material

Screening of metabolism-disrupting chemicals on pancreatic α-cells using in vitro methods

Supplementary Table S1. List of antibodies used in this study.

**Target antigen**	**Antibody Name**	**Manufacturer and catalogue number (Cat no.)**	**Species raised in**	**Dilution**	**RRID**
BiP	BiP Antibody	Cell Signaling Technology; Cat no. 3183	Rabbit, polyclonal	1:1000	AB_668355
p-eIF2α	Phospho-eIF2α (Ser51) (119A11)	Cell Signaling Technology; Cat no. 3597	Rabbit, monoclonal	1:1000	AB_390740
α-Tubulin	Monoclonal Anti-αTubulin antibody	Sigma; Cat no. T9026	Mouse, monoclonal	1:5000	AB_477593
Goat anti-mouse IgG	Goat Anti-Mouse IgG (H+L) HRP Conjugate antibody	Bio-rad; Cat no. 170-6516	Goat, Polyclonal	1:5000	AB_11125547
Goat anti-rabbit IgG	Goat Anti-Rabbit IgG (H+L) HRP Conjugate antibody	Bio-rad; Cat no. 170-6515	Goat, Polyclonal	1:5000	AB_11125142

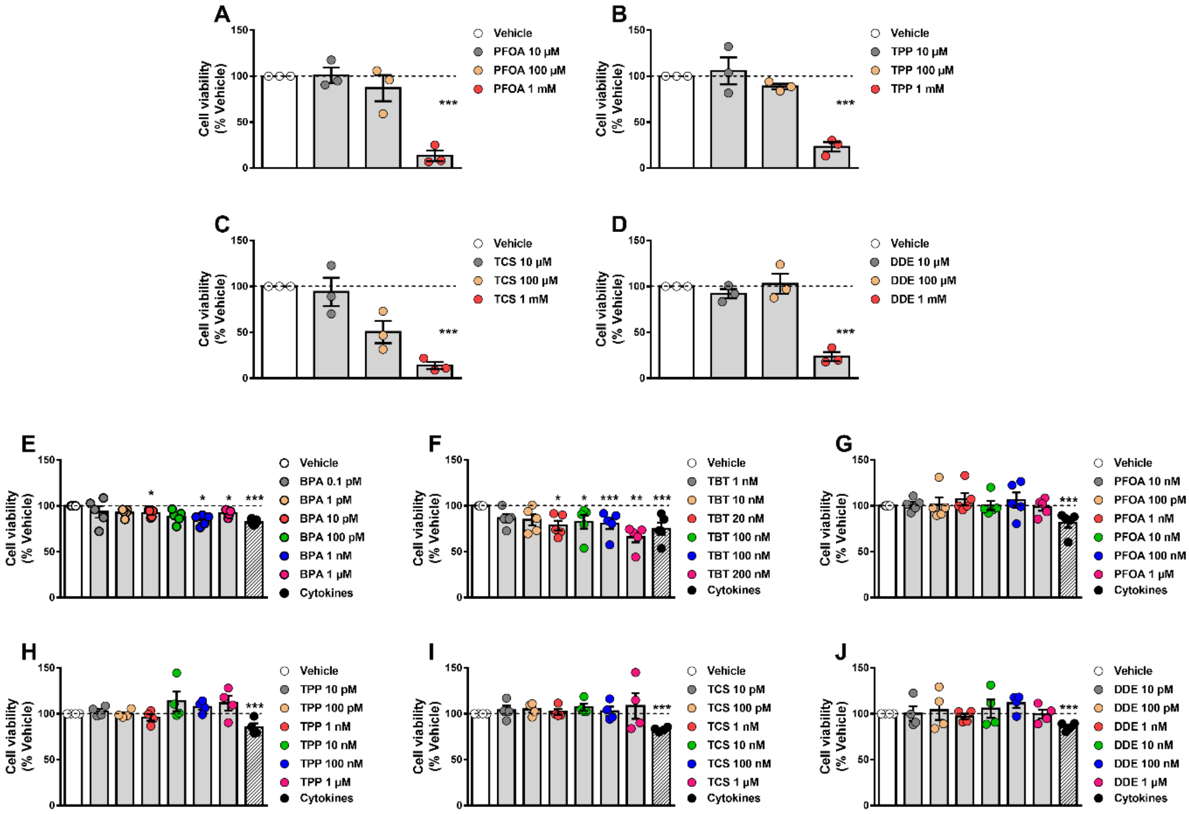

Supplementary Figure S1. α-cell viability upon MDC exposure. (A-D) αTC1-9 cells were treated with vehicle (DMSO) or different doses of PFOA (A), TPP (B), TCS (C), or DDE (D) for 48 h. (E-J) αTC1-9 cells were treated with vehicle (DMSO) or different doses of BPA (E), TBT (F), PFOA (G), TPP (H), TCS (I), or DDE (J) for 72 h. A cocktail of the cytokines IL-1β + IFNγ (50 and 1000 U/ml, respectively) was used as a positive control. Cell viability was evaluated by MTT assay. Results are expressed as % vehicle-treated cells. Data are shown as means ± SEM (n = 3-5 independent experiments, where each dot represents an independent experiment). * $p \leq 0.05$, ** $p \leq 0.01$ and *** $p \leq 0.001$ vs. Vehicle. MDCs vs. Vehicle by one-way ANOVA; Cytokines vs. Vehicle by two-tailed Student's *t* test.
